# Computational study on the haemodynamic and mechanical performance of electrospun polyurethane dialysis grafts

**DOI:** 10.1007/s10237-019-01242-1

**Published:** 2019-11-02

**Authors:** Sjeng Quicken, Yeshi de Bruin, Barend Mees, Jan Tordoir, Tammo Delhaas, Wouter Huberts

**Affiliations:** 1 Department of Biomedical Engineering, CARIM School for Cardiovascular Diseases, Maastricht University, Universiteitssingel 50, 6223ER Maastricht, The Netherlands; 2 Department of Biomedical Engineering, Eindhoven University of Technology, PO Box 513, 5600 MB Eindhoven, The Netherlands; 3 Department of Vascular Surgery, Maastricht University Medical Centre, PO Box 5800, 6229 HX Maastricht, The Netherlands

**Keywords:** Fluid structure interaction modelling, Dialysis graft, Material choice, Polyurethane

## Abstract

Compliance mismatch between an arteriovenous dialysis graft (AVG) and the connected vein is believed to result in disturbed haemodynamics around the graft–vein anastomosis and increased mechanical loading of the vein. Both phenomena are associated with neointimal hyperplasia development, which is the main reason for AVG patency loss. In this study, we use a patient-specific fluid structure interaction AVG model to assess whether AVG haemodynamics and mechanical loading can be optimised by using novel electrospun polyurethane (ePU) grafts, since their compliance can be better tuned to match that of the native veins, compared to gold standard, expanded polytetrafluoroethylene (ePTFE) grafts. It was observed that the magnitude of flow disturbances in the vein and the size of anastomotic areas exposed to highly oscillatory shear ($\hbox {OSI} >0.25$) and very high wall shear stress ($>40 \hbox { Pa}$) were largest for the ePTFE graft. Median strain and von Mises stress in the vein were similar for both graft types, whereas highest stress and strain were observed in the anastomosis of the ePU graft. Since haemodynamics were most favourable for the ePU graft simulation, AVG longevity might be improved by the use of ePU grafts.

## Introduction

Haemodialysis (HD) is the main mode of renal replacement therapy for most patients with end-stage renal disease. During HD, a haemodialyser removes metabolic waste products and excess fluids from the patient’s blood stream. A functional vascular access (VA) is of vital importance for these patients, since this is the only location where the haemodialyser can be connected to the body during HD. VAs need to be created surgically, as no autologous vessel allows for easy and repeated cannulation, whilst also providing the high blood flow ($>500 \hbox { ml/min}$) required for efficient haemodialysis. Autologous arteriovenous fistulas (AVF) are the preferred type of VA as they show the lowest complication rates and superior long-term patencies with respect to available alternatives (Tordoir et al. 2007). However, as a result of insufficient vessel quality, not all patients are suitable for an autologous access. Consequently, a significant number of patients, amounting to $18\%$ and $7\%$ of the total haemodialysis population in the USA (USRDS 2017) and Europe (Noordzij et al. 2014), respectively, rely on a synthetic arteriovenous graft (AVG) for receiving HD treatment. This percentage might increase in the future, as recent research suggests that, for patients with limited life expectancy, AVGs show fewer complications compared to AVFs (Hall et al. 2017; Lee et al. 2018).

The majority of currently used AVGs are made from expanded polytetrafluoroethylene (ePTFE). Despite multiple interventions to maintain patency, these grafts have a typical lifespan of only 2 years. This short lifetime of grafts can mainly be attributed to venous neointimal hyperplasia (NIH) near the graft–vein anastomosis, resulting in stenosis, low flow and ultimately thrombosis and graft patency loss. Disturbed flow and resulting non-physiological or multi-directional wall shear stress (WSS) after AVG creation, as well as limited biocompatibility are believed to be the main triggers of NIH development (Lee and Haq 2015). Furthermore, it has been hypothesised that mismatch in mechanical properties between the vein and graft (i.e. compliance mismatch) plays an important role in graft dysfunction by further promoting disturbed flow and causing excessive pulsatile loading of the veins (Abbott et al. 1987; Hofstra et al. 1994).

Most research on optimising AVGs has focussed on the development of novel, haemodynamically optimised, graft designs (Moufarrej et al. 2016). Strikingly, even though ePTFE grafts are up to 400–500 times stiffer than native veins (Catanese et al. 1999; Huberts et al. 2012), the effect of minimising graft–vein compliance mismatch on anastomotic haemodynamics and mechanical loading of the vein is relatively unexplored in the pursuit of longer lasting grafts. Because both large inter-patient variation (Halliwill et al. 1999) and the effect of vascular remodelling hamper the prediction of vessels’ mechanical properties after AVG creation, development of a graft that completely eliminates compliance mismatch is difficult and not very likely in the near future. However, the use of more compliant graft materials can considerably reduce compliance mismatch.

Electrospun polyurethane (ePU) has emerged as a possible substitute to ePTFE for vascular grafts (Hu et al. 2012). Since ePU can be manufactured over a large range of mechanical properties (Montini-Ballarin et al. 2016), resulting grafts can be tuned to better match the mechanical properties of native veins, thereby minimising graft–vein compliance mismatch. Furthermore, since the grafts are spun, they exhibit a fibrous structure that closely resembles native extracellular matrix (Hu et al. 2012). This structure allows for better graft endothelialisation compared to ePTFE grafts, which enhances the graft’s biocompatibility (Hu et al. 2012). Early small-cohort trials have furthermore demonstrated the efficacy of ePU grafts for dialysis access (Ferraresso et al. 2013; Karatepe et al. 2013; Wijeyaratne and Kannangara 2011; Yilmaz 2016).

The aim of this study was to assess whether reducing graft–vein compliance mismatch, by using ePU grafts instead of ePTFE grafts, could help improve anastomotic AVG haemodynamics and mechanical behaviour. Patient-specific fluid-structure interaction (FSI) models of the venous anastomosis of an ePTFE and an ePU graft were developed, that allowed for studying the impact of different graft materials on blood flow. In this respect, the modelling approach differs from more commonly applied computational fluid dynamics models that assume rigid vessels. Furthermore, haemodynamic and mechanical properties that would be hard to measure in experimental set-ups, could be straightforwardly computed from the FSI model.

## Materials and methods

The patient data used in this study were obtained from clinical follow-up data for monitoring graft function and was measured at the Maastricht University Medical Centre (*Maastricht, the Netherlands*). A waiver for ethical approval for this study was obtained from the local medical ethical committee.

### FSI model

#### Anastomotic model

**Fig. 1:**
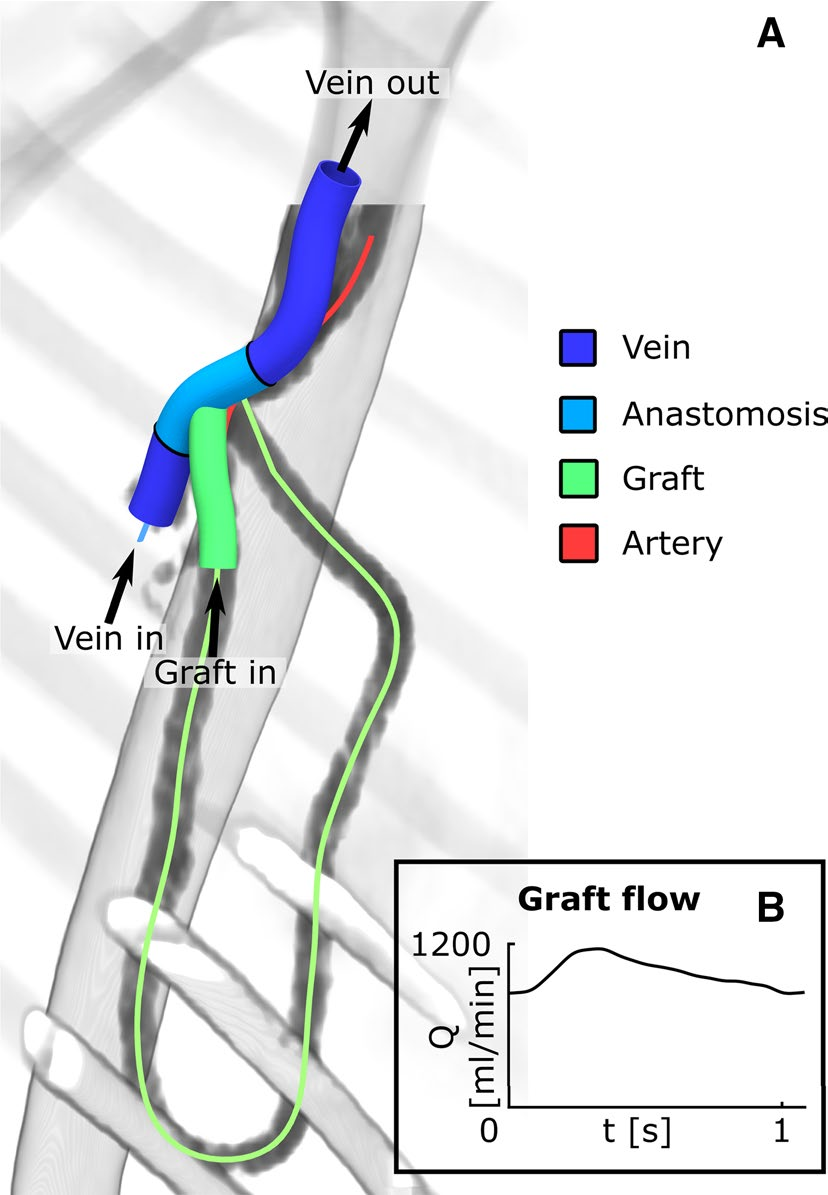
**a** Overview of the AVG geometry and regions of interest, imposed on the CTA data and the extracted vessel centrelines. Blood flow orientation is indicated by the arrows. Note that blood flow velocity at the venous inlet was set to zero. **b** Prescribed flow (Q) at the graft inlet

A patient-specific axillary-artery to axillary-vein loop AVG geometry was reconstructed from 15-month post-operative diagnostic computed tomography angiography (CTA) data and 2-week pre-operative ultrasound diameter measurements of a single patient (Fig. 1a). It was assumed that, though vessel diameter could have changed between AVG surgery and CTA imaging, vessel path would have remained relatively unchanged. The 15-month post-operative AVG geometry was obtained by segmentation of the CTA images using the software package VMTK (Antiga et al. 2008). Centrelines of the geometry were extracted to define vessel shape and graft configuration. After importing the centrelines into SolidWorks (*2018, Dassault Systèmes, Vélizy-Villacoublay, France*), vessels with a circular cross section were imposed onto the centrelines, representing the inner lumen of graft and the vein at zero transmural pressure. Zero-pressure graft diameter was set to 6 mm, whereas zero-pressure vein diameter was reconstructed from pre-operative ultrasound data and was set to $7.14 \hbox { mm}$ (“Appendix A”). Wall thickness of the vein was assumed to be $10\%$ of its pre-operative radius and graft wall thickness was set to $0.63 \hbox { mm}$. To reduce the computational cost of the simulations, the graft and venous segments were trimmed to $3 \hbox { cm}$ and $7.5 \hbox { cm}$, respectively. The anastomosis was located at approximately 1/3th of the venous segment.

Geometries were meshed in ICEM CFD (*17.2, Ansys, Canonsburg, PA, USA*). A mesh independent solution was obtained for $2.5 \cdot 10^{6}$ and $0.6\cdot 10^{6}$ elements in the fluid and the solid domain, respectively.

#### Boundary conditions

Graft flow was assessed approximately seven weeks after AVG surgery and was imposed at the graft inlet by prescribing a pulsatile parabolic velocity profile (Fig. 1a, b). Flow from the peripheral veins was assumed to be negligible compared to the graft flow and, hence, blood flow velocity was set to zero on the venous inlet boundary. A resistive boundary condition was prescribed at the model’s venous outlet to ensure an average proximal venous pressure of approximately 50 mmHg (Van Tricht et al. 2004). A no-slip condition was enforced on the blood-vessel interface. Finally, the in- and outlets of the model were fixed in space to prevent migration of the model, whereas extravascular pressure was set to 0 mmHg.

#### Computational model and material properties

Simulations were performed using the unified-continuum, arbitrary Lagrange–Eulerian (ALE) FSI solver Unicorn (Hoffman et al. 2013), implemented in the finite-element package FEniCS (Logg et al. 2012). Blood and vessel walls were considered as a single continuum, subjected to the same governing equations for mass and momentum balance, albeit with different constitutive laws dictating material behaviour. Spatial discretisation was achieved using piecewise linear tetrahedral elements for both pressure and velocity. An implicit Crank-Nicolson scheme was used to progress the simulations in time (Hoffman et al. 2013). Stepping size was set to $1\times 10^{-4}\hbox { s}$, which was reduced to $7\cdot 10^{-5}\hbox { s}$ over a quarter-second interval during peak systole to be able to better resolve the higher flow velocities.

Blood flow was modelled as an incompressible Newtonian fluid with a dynamic viscosity of $4.5\cdot 10^{-3}\hbox { Pa s}$ (Dhar et al. 2012) and density of $1000 \hbox { kg m}^{-3}$. Flow was numerically stabilised using a streamline diffusion method (Hoffman et al. 2013). Flow was considered laminar because the effect of adding a turbulence model had a negligible effect on the computed output metrics (non-published results).

Since data on the mechanical behaviour of veins and grafts are scarce, mechanical behaviour of the vessel walls was characterised with an incompressible neo-Hookean material law. The axillary vein was assumed to have similar properties as the cephalic vein and its Young’s modulus was set to 0.445 MPa, which was obtained by linearising the cephalic vein stress-strain behaviour (unpublished data) in the post-operative working range 30–60 mmHg (Van Tricht et al. 2004). The Young’s modulus of the ePTFE and ePU grafts were set to 55 MPa (Catanese et al. 1999) and 1.5 MPa (Montini-Ballarin et al. 2016), respectively.

Simulations performed on a single node of the Cartesius supercomputing cluster (*SURFsara, the Netherlands*) consisting of 24 CPU cores took approximately 15 days to complete.

### Haemodynamic and mechanical metrics

All mechanical and haemodynamic metrics of interest were computed over the second simulated cardiac cycle. Mechanical and haemodynamic outputs of interest were computed both over the total simulated venous segment and, more locally, in the venous anastomotic region, which was defined as the venous region encapsulated in a spherical domain around the anastomosis, with a radius of 1.2 cm (Fig. 1a). Furthermore, mechanical metrics were also computed for the graft segment (Fig. 1a).

Though WSS is considered to play an important role in AVG dysfunction, the exact WSS metric that triggers NIH development is unknown (Cunnane et al. 2017). In this study, it was assumed that WSS outside the physiological range of 0.1–7.0 Pa (Malek et al. 1999) would be detrimental to AVG function. Furthermore, since WSS in excess of 40 Pa can cause irreversible endothelial damage within one hour of exposure (Fry 1968), any region where this threshold was exceeded was also identified. Finally, regions exposed to highly oscillatory WSS were assessed.

The region size exposed to non-physiologically low WSS ($<0.1$ Pa) was determined using the time-averaged wall shear stress magnitude (TAWSS):

1 $$\begin{aligned} \text {TAWSS} = \frac{1}{T}\int _0^T \left\| \varvec{\tau }(t,\mathbf {x})\right\| \mathrm{{d}}t, \end{aligned}$$

where $\varvec{\tau }(t,\mathbf {x})$ represents the WSS vector and *T* the duration of the cardiac cycle. Exposure to non-physiologically high WSS ($>7$ Pa) and very high WSS ($>40$ Pa) was assessed by computing the time-maximum WSS ($\text {WSS}_{\text {max}}$):

2 $$\begin{aligned} \text {WSS}_{\text {max}} = {\text {max}}\left\{ \left\| \varvec{\tau }(t, \mathbf {x})\right\| :t = 0..T \right\} . \end{aligned}$$

Oscillatory behaviour of the wall shear stress was quantified by the oscillatory shear index (OSI) (He et al. 1996):

3 $$\begin{aligned} \text {OSI} = \frac{1}{2}\left( 1 - \frac{\left\| \int _0^T\varvec{\tau }(t, \mathbf {x})\mathrm{{d}}t\right\| }{\int _0^T\left\| \varvec{\tau }(t, \mathbf {x})\mathrm{{d}}t\right\| } \right) , \end{aligned}$$

which ranges between 0.0 and 0.5. An OSI $>0.25$ was used as a threshold for highly oscillating WSS.

The magnitude of (turbulent) velocity perturbations in the vein was quantified using Reynolds decomposition. Here, flow velocity magnitude $u(t,\mathbf {x})$ was decomposed into the sum of the average velocity trend ${\bar{u}}(t,\mathbf {x})$ and high-frequency velocity perturbations ${\tilde{u}}(t,\mathbf {x})$. Finally, ${\tilde{u}}_{\text {RMS}}(\mathbf {x})$ was computed as the root mean square value of ${\tilde{u}}(t, \mathbf {x})$ over the cardiac cycle, which was used to quantify the amount of disturbed flow at any point in the vein.

Mechanical stress in venous and graft walls was characterised by the von Mises stress:

4 $$\begin{aligned} \sigma _\text {M} = \sqrt{\frac{3}{2}{\text {tr}}(\varvec{\sigma }_d \cdot \varvec{\sigma }_d)}, \end{aligned}$$

a scalar stress measure, based on the deviatoric stress tensor $\varvec{\sigma }_d$, representing the equivalent stress if strictly one-dimensional loading would be applied to the material. Wall strain was computed over the inner and outer surface of the graft wall according to:

5 $$\begin{aligned} \varepsilon = \sqrt{\frac{A_n - A_0}{A_0}}. \end{aligned}$$

Here, $A_0$ and $A_n$ represent the initial area and the area during simulation of a surface element. The metric $\varepsilon$ is equivalent to the definition of engineering strain and lumps circumferential and longitudinal strain in one metric. Pulsatility in $\sigma _M$ and $\varepsilon$ was computed for each mesh element by computing the difference between the minimum and the maximum value observed during the analysed cardiac cycle. Reported maxima for all wall shear stress, von Mises stress and strain measures, were computed after omitting the $1\%$ region with the highest values to exclude any simulation artefacts.

## Results

### Haemodynamic AVG behaviour

**Fig. 2:**
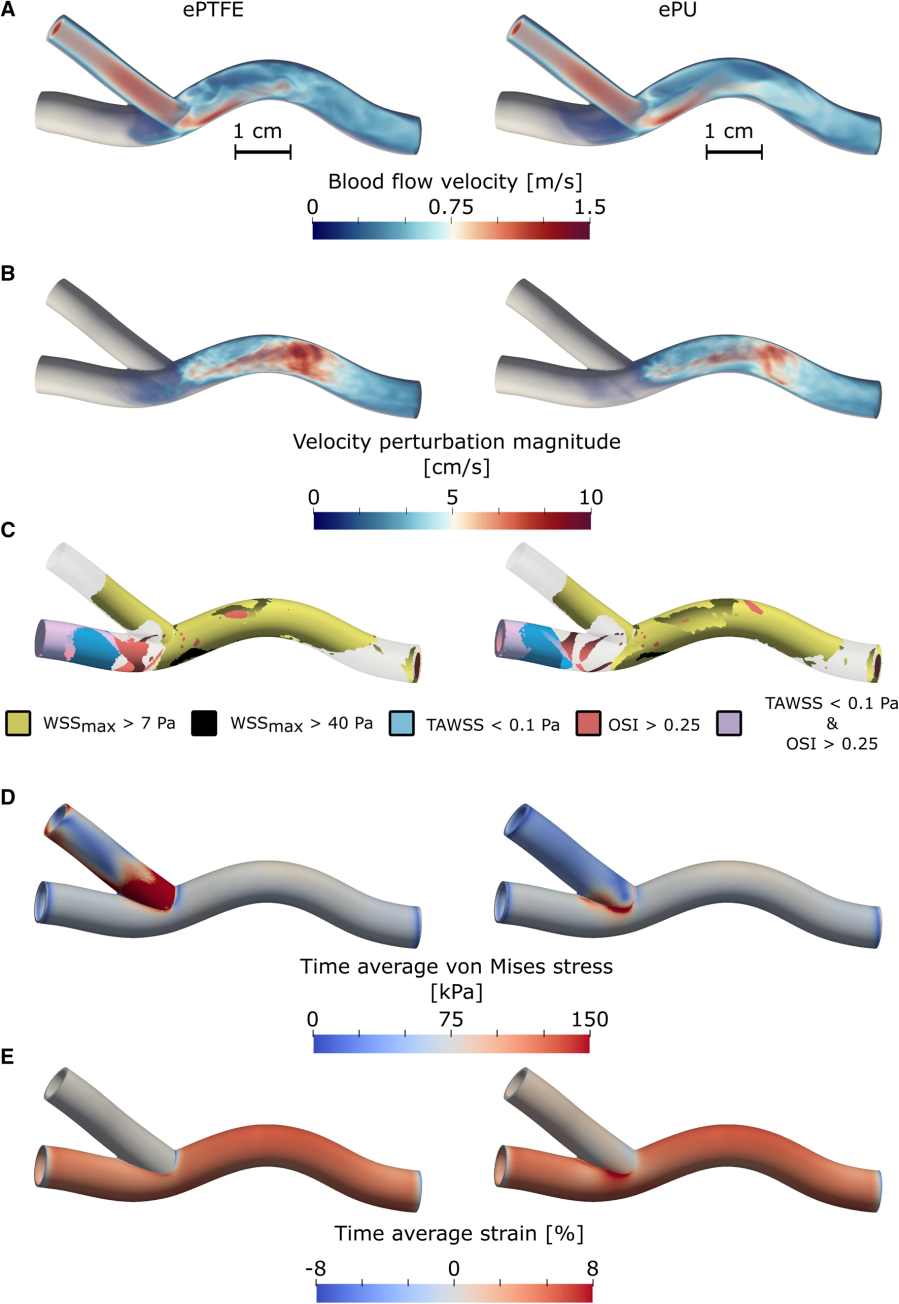
Blood flow velocities at peak systole (**a**), magnitude of (turbulent) velocity perturbations (**b**), location and size of WSS regions of interest (**c**), time average von Mises stress $\sigma _\text {M}$ (**d**) and the time average strain $\varepsilon$ (**e**) in the ePTFE (left) and ePU (right) graft simulations. All variables are plotted onto the reference geometry, except the blood flow velocity, which is plotted onto the geometry at peak systole. For both volume renders presented in **a** and **b**, opacity is scaled by the local magnitude of the plotted variable. Note that since the magnitude of velocity perturbations are projected onto the zero-stress reference geometry, outward movement of the curved segment of the geometries can be observed by comparison of the geometries presented in pane **a** (peak systole), with those presented in pane **b**–**e**. The colour maps in **a**, **b** and **d**, **e** were chosen differently to optimise visibility for volume or surface rendering, respectively

**Fig. 3:**
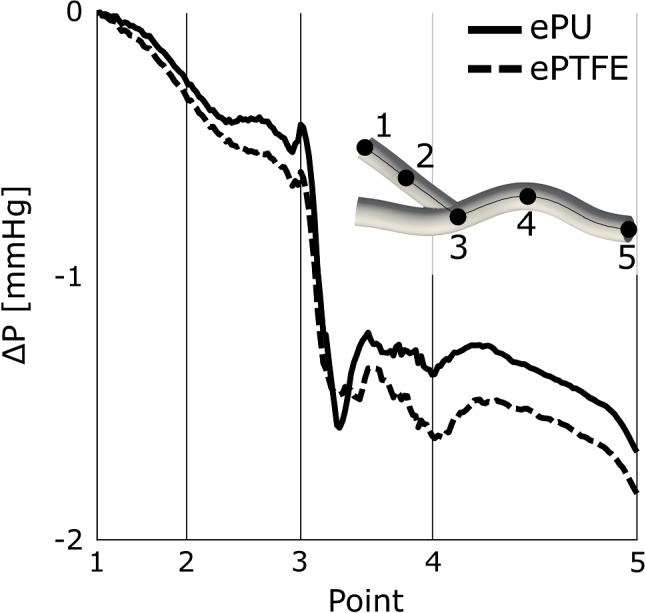
The time-averaged pressure drop over the centreline from the graft’s inlet to the venous outlet for both the ePU and the ePTFE AVG geometry. Note that the points on the plot’s horizontal axis of coincide with the points indicated in the AVG geometry on the right

Flow in the graft was laminar during the complete cardiac cycle for both the ePTFE and the ePU graft simulations. After leaving the graft, flow impinged on the venous wall, giving rise to secondary flow and a jet along the venous floor (Fig. 2a). In both simulations, higher flow velocities were observed in the graft segment compared to the vein. Maximum flow velocity in the graft was slightly higher for the ePTFE graft simulation ($1.44 \hbox { m s}^{-1}$) compared to the ePU graft ($1.41 \hbox { m s}^{-1}$). The time-averaged pressure drop from the AVG model’s arterial inlet to the venous outlet was similar for both the ePU and ePTFE graft and ranged between 1.7 and 1.8 mmHg (Fig. 3). At the venous anastomosis (Fig. 3, between point 3 and 4), the pressure of the ePU graft experienced a large drop, followed by a partial pressure recovery. Though the immediate pressure drop at the venous anastomosis of the ePTFE graft was smaller, no similar pressure recovery was observed.

The magnitude of velocity perturbations in the venous segment was generally higher in the ePTFE graft compared to that of the ePU graft (Fig. 2b). In both simulations, the median value of the velocity perturbation magnitude was less than $1 \hbox { cm s}^{-1}$ at the anastomotic site and increased to its maximum value at the curved segment of the vein (cross-sectional median velocity perturbations of $4.9\hbox { cm s}^{-1}$ and $5.5\hbox { cm s}^{-1}$ for the PU and the ePTFE model, respectively). As a result from pressurising the vessel, the curve in the venous segment moved outward by maximally 2.5 mm (Fig. 2). During the cardiac cycle, outward movement of the curved segment was within 0.7 mm.

Both the anastomotic and the total venous area that were exposed to highly oscillatory shear (OSI $>0.25$) were approximately twice as large in the ePTFE graft simulation compared to the ePU graft simulation (Table 1). Large regions of high OSI were observed at the distal venous boundary and in the distal part of the anastomosis of both simulations. Smaller regions with high oscillatory shear were observed near the anastomotic toe and in the curved segment of the vein (Fig. 2c). Peak OSI in the anastomosis was higher for the ePTFE graft (0.44) compared to the ePU graft (0.38).

Non-physiologically low TAWSS ($<0.1$) was almost absent in the anastomotic region of either graft (Table 1). The total venous area subjected to low TAWSS was $19\%$ and $16\%$ for the ePTFE and the ePU graft, respectively (Table 1). The area with low TAWSS was mainly located distal to the venous anastomosis (Fig. 2c). Median TAWSS in the vein differed slightly for the ePTFE (3.5 Pa) and ePU (3.1 Pa) graft.

Time-maximum WSS was substantially higher in the region with graft flow impingement on the venous floor, compared to the rest of the vein. The area exposed to non-physiologically high WSS ($>7$ Pa) was approximately $60\%$ of the anastomotic region for both graft types (Table 1 and Fig. 2c). Though anastomotic area subjected to very high WSS ($>40$ Pa) was small for both graft types, it was roughly 2.5 times larger for ePTFE graft compared to the ePU graft (Table 1, Fig. 2c). Finally, maximum WSS in the anastomosis of the ePTFE graft was approximately $10\%$ higher (46.5 Pa) compared to maximum WSS in the anastomosis of the ePU graft (41.8 Pa).

**Table 1:** Overview of exposure to WSS metrics in the regions of interest of both graft types

	Vein		Anastomosis	
	ePTFE	ePU	ePTFE	ePU
OSI $>0.25$ ($\%$)	13.5	7.6	11.9	5.4
TAWSS $<0.1$ Pa ($\%$)	18.6	16.1	0.2	0.0
$$\text {WSS}_\text {max}$$$$>7$$ Pa ($$\%$$)	47.9	48.2	61.4	58.6
$$\text {WSS}_\text {max}$$$$>40$$ Pa ($$\%$$)	1.2	0.5	4.2	1.7

### Mechanical AVG behaviour

For both simulations, highest von Mises stress ($\sigma _M$) was observed in the graft near the anastomosis (Fig. 2d). Median and maximum stress, as well as stress pulsatility were considerably higher in the ePTFE graft, compared to the ePU graft (Table 2). Median stress and stress pulsatility in the vein were similar for both graft types. Maximum stress in the anastomotic region of the ePU graft simulation was almost 1.5 times higher than that of the ePTFE graft (Table 2). Furthermore, higher peak pulsatility was observed in the anastomotic region of the ePU graft compared to that of the ePTFE graft (Table 2).

**Table 2:** Overview of von Mises stress metrics in the regions of interest of both graft materials

	Graft		Vein		Anastomosis	
	ePTFE	ePU	ePTFE	ePU	ePTFE	ePU
Median $\sigma _\text {M}$ (kPa)	75	40	71	72	72	75
Median pulsatility $\sigma _\text {M}$ (kPa)	25	12	24	24	24	25
Maximum $\sigma _\text {M}$ (kPa)	477	146	82	96	84	120
Maximum pulsatility $\sigma _\text {M}$ (kPa)	151	40	28	30	28	35

The ePTFE graft showed negligible strain during the cardiac cycle (Table 3). Median strain in the ePU graft was under $1\%$, but small regions exposed to more than $3\%$ were observed close to the venous anastomosis (Fig. 2e). Median strain in the veins of both simulations were similar, but maximum strain was approximately $30\%$ higher around the venous anastomosis of the ePU graft compared to the anastomotic region of the ePTFE graft. Median venous and anastomotic strain pulsatility were similar for both graft types (Table 3).

**Table 3:** Overview of strain metrics in the regions of interest of both graft materials

	Graft		Vein		Anastomosis	
	ePTFE	ePU	ePTFE	ePU	ePTFE	ePU
Median $\varepsilon$ ($\%$)	0.0	0.8	4.7	4.9	4.9	5.2
Median pulsatility $\varepsilon$ ($\%$)	0.0	0.3	1.5	1.6	1.6	1.7
Maximum $\varepsilon$ ($\%$)	0.3	3.6	6.3	6.5	5.9	7.8
Maximum pulsatility $\varepsilon$ ($\%$)	0.1	0.9	2.0	2.1	1.9	2.1

## Discussion

Disturbed haemodynamics are believed to be the main trigger in the development of venous NIH and consequent AVG dysfunction. The aim of this study was to elucidate whether AVG haemodynamics can be improved by material selection. More specifically, we compared haemodynamic and mechanical performance of a gold standard ePTFE graft with a novel ePU graft. The latter graft material has emerged as a possible alternative to ePTFE; it can be tuned to better match the mechanical properties of veins and shows excellent biocompatibility (Hu et al. 2012). This is the first *in silico* study to compare the impact of different AVG materials on haemodynamic and mechanical graft performance.

It was demonstrated that graft material selection has a considerable impact on the amount of disturbed flow as well as the amount of non-physiological and oscillatory wall shear stress in the direct vicinity of the venous anastomosis of AVGs. Velocity disturbances in the vicinity of the anastomosis of the ePU AVG geometry were substantially lower in magnitude as those observed around the anastomosis of the ePTFE AVG geometry. Furthermore, the total anastomotic area exposed to highly oscillatory wall shear stress or to wall shear stress in excess of 40 Pa was considerably smaller in the anastomosis of the ePU graft. Besides an overall reduction of the OSI and an increase in maximum von Mises stress over the total venous region of the ePU AVG, the impact of graft material on venous haemodynamics and mechanical loading diminished at larger distances from the anastomosis.

The observation that graft mechanical behaviour has a large impact on venous anastomotic haemodynamics is in line with the hypothesis that a considerable graft–vein compliance mismatch induces disturbed flow (Abbott et al. 1987; Hofstra et al. 1994). Alternatively, the differences in haemodynamic properties might be explained by the increased compliance of the ePU graft with respect to the ePTFE graft alone, regardless of its reduced compliance mismatch with the veins. It was expected (and observed) that ePTFE showed less distension compared to the ePU graft, given a similar load, due to its higher Young’s modulus. Consequently, for a similar load, cross-sectional graft area is larger for an ePU graft than for an ePTFE graft, allowing for lower blood flow velocities given the same flow rate. Indeed, maximum flow velocities were lower in the ePU graft, compared to those observed in the ePTFE graft. Since both the presence of turbulent velocity perturbations and the magnitude of observed wall shear stresses are directly dependent on blood flow velocity, the difference in observed haemodynamics might be solely attributed to the increased compliance of ePU grafts rather than to the decreased graft–vein compliance mismatch. Similarly, for arteriovenous fistulas, it has been observed that anastomotic blood flow velocities (Decorato et al. 2014) and WSS (Decorato et al. 2014; McGah et al. 2014) were significantly higher in simulations that assumed completely non-compliant (rigid) arteriovenous fistulas, compared to simulations that included fistula compliance. Developing a graft that minimises graft–vein compliance mismatch is challenging because of the large inter-patient variability in venous mechanical properties (Halliwill et al. 1999). If graft compliance, rather than graft–vein compliance, mismatch is indeed a more important parameter for obtaining favourable haemodynamics, it might not be necessary to pursue a graft with mechanical properties that exactly match those of the autologous veins. Instead, the use of a more compliant graft material, such as ePU, could already have an important contribution to increasing graft longevity. Nevertheless, future studies should be performed to verify this hypothesis and establish whether graft performance can be improved by further increasing graft compliance.

AVG function is for a large part dependent on the amount of blood flow through the graft. Consequently, flow resistance of the ePU graft is ideally similar to that of an ePTFE graft to prevent a too high or too low blood flow that can, for instance, cause steal syndrome and insufficient dialysis flow, respectively. Since blood flow for both AVG models was equal, similar pressure drops over the simulated domains of both graft types indicate that graft impedance at the venous anastomosis is minimally affected by the choice of graft material. However, since the largest pressure drop occurs at the graft’s arterial anastomosis (Van Tricht et al. 2004), future studies should evaluate how graft material impacts the blood flow resistance of the total AVG geometry.

Median mechanical loading of the veins was similar for both graft types. Maximum stresses and strains, as well as stress and strain pulsatility, were however higher around the anastomosis of the ePU graft. A possible explanation for the observed behaviour is that the veins connected to the ePTFE graft are more constraint in space, as ePTFE does not distend itself. As such, it protects the vein to extensive straining. On the other hand, the ePU graft does show some distension during the cardiac cycle and thus does not limit the movement of the venous wall as much. Since stress and strain are dependent, the increased stress around the anastomosis of the ePU graft is a direct consequence of the increased venous strain. A similar observation was made by Hofer et al. (1996), who found increased displacement around the anastomosis of end-to-side anastomosis models with more compliant grafts.

Although the exact biological response is not completely understood, both disturbed flow and non-physiological wall shear stress are believed to play an important role in neointimal hyperplasia development and subsequent AVG patency loss (Lee and Haq 2015). Furthermore, pulsatile stretch has been shown to result in proliferation of venous smooth muscle cells (Predel et al. 1992), an important factor in the development of NIH. In a recent *in vitro* study, however, the effect of pulsatile stretch on the proliferation of human venous cells has been reported to be overruled in the presence of WSS (van Haaften et al. 2018). Consequently, we believe that, in comparison to the observed haemodynamic effects, the higher von Mises stresses and strains observed in the anastomotic region of the ePU graft compared to the ePTFE graft have limited impact on the longevity of ePU grafts.

Since the area exposed to non-physiological WSS and the amount of disturbed flow in the vicinity of the venous anastomosis could be reduced with the use of an ePU graft, the results of this study suggest that ePU grafts might show better long-term patency compared to ePTFE grafts. Though currently no large trials have been performed to support this statement, several small-cohort clinical trials evaluating a multi-layer ePU haemodialysis graft reported slightly better (Karatepe et al. 2013) or comparable (Ferraresso et al. 2013; Wijeyaratne and Kannangara 2011; Yilmaz 2016) patencies to ePTFE grafts. Since the ePU grafts used in the latter studies are of the same multi-layer graft design, of which only one layer was specified to mimic the elastic properties of native blood vessels (Ferraresso et al. 2013; Wijeyaratne and Kannangara 2011), it is unclear to what extent the complete graft matches the biomechanical properties of native vessels, and thus, to what extent the results can be attributed to increased graft compliance.

### Limitations

In this study, all vessels were modelled using a neo-Hookean material model that requires the estimation of only one mechanical parameter per material. Although, opposed to the neo-Hookean model, more elaborate models (Holzapfel et al. 2002; Rezakhaniha and Stergiopulos 2008) allow for modelling the anisotropic and viscoelastic material behaviour observed in blood vessels and grafts (Montini-Ballarin et al. 2016; Rezakhaniha and Stergiopulos 2008), these models also require the estimation of more constitutive model parameters. Since data on the mechanical behaviour of veins (especially at high pressures) and grafts are scarce, estimation of these constitutive parameters is hard and is hampered by a large uncertainty. However, because mechanical properties of ePTFE and ePU are vastly different, we believe that, despite the use of a neo-Hookean model, the results obtained in this study do give a good insight into the different haemodynamic and mechanical behaviour of ePTFE and ePU grafts. Nevertheless, future work should investigate to what extent results are influenced by the material model choice.

Furthermore, in this study, we modelled a geometry with relatively short graft and vein segments to reduce computational cost. Consequently, volume storing capacity, and therefore compliance of the grafts, was relatively low, possibly impacting flow in the venous anastomosis. However, because compliance difference between ePTFE and ePU grafts will only become larger with longer vessel segments, we believe that the differences in haemodynamic performance of both graft types will become even more pronounced with longer vessel segments.

In this study, the blood-vessel path was patient-specifically reconstructed. However, because AVGs are typically only considered in patients whose vessel quality is insufficient for the creation of an autologous arteriovenous fistula, the assumption of circular cross-sectional blood vessels could be too idealised for this patient-population. Future research should assess how the assumption of circular cross-sectional vessels impact haemodynamics.

## Conclusion

In this study, we demonstrated that, compared to gold standard expanded polytetrafluoroethylene (ePTFE), the magnitude of flow disturbances and the size of the anastomotic area exposed to very high or oscillatory wall shear stress can be considerably reduced by using more compliant electrospun polyurethane (ePU) grafts. Since AVG failure induced by neointimal hyperplasia is triggered by disturbed blood flow and non-physiological wall shear stress, the results from this study suggest that AVG longevity will be improved by the use of ePU grafts. The improved *in vivo* performance of novel, compliant ePU grafts should be evaluated in future studies.

## Appendix A: Zero-pressure vein diameter

Axillary vein diameter under physiological venous pressure [$\approx 10 \hbox { mmHg}$ (Delaney et al. 2008; Halliwill et al. 1999)] was preoperatively measured as 7.7 mm using ultrasound. Zero-pressure vein diameter was estimated using Laplace’s law, which assumes a linear stress-strain relation and a wall thickness that is much smaller than the vessel’s inner radius.

Laplace’s law is given by:

6 $$\begin{aligned} \partial x_\mathrm{{r}} = \frac{p a_0^2}{h E}(1-\nu ^2), \end{aligned}$$

and relates radial displacement $\partial x_\mathrm{{r}}$ to the internal pressure *p*, the zero-pressure inner radius $a_0$, the wall thickness *h* and the material’s Young’s modulus *E* and Poisson’s ratio $\nu$. Radial displacement can be rewritten as:

7 $$\begin{aligned} \partial x_\mathrm{{r}} = a_{10} - a_0, \end{aligned}$$

where $a_{10}$ represents the measured radius at 10 mmHg. Combining Eqs. (6) and (7) results in:

8 $$\begin{aligned} \frac{p(1-\nu ^2)}{h E} a_0^2 + a_0 - a_{10} = 0 \end{aligned}$$

Under physiological pressures, the Young’s modulus of the axillary vein was assumed to be similar to that of the cephalic vein: 0.12 MPa (Huberts et al. 2012). The vein was assumed to be incompressible, resulting in a Poisson’s ratio of 0.5. Wall thickness was assumed as $10\%$ of the radius under physiological pressure (0.385 mm). Solving Eq. (8) for $a_0$ results in an internal diameter of 7.14 mm.

## References

[CR1] Abbott WM, Megerman J, Hasson JE, L’Italien G, Warnock DF (1987). Effect of compliance mismatch on vascular graft patency. J Vasc Surg.

[CR2] Antiga L, Piccinelli M, Botti L, Ene-Iordache B, Remuzzi A, Steinman DA (2008). An image-based modeling framework for patient-specific computational hemodynamics. Med Biol Eng Comput.

[CR3] Catanese J, Cooke D, Maas C, Pruitt L (1999). Mechanical properties of medical grade expanded polytetrafluoroethylene: the effects of internodal distance, density, and displacement rate. J Biomed Mater Res.

[CR4] Cunnane CV, Cunnane EM, Walsh MT (2017). A review of the hemodynamic factors believed to contribute to vascular access dysfunction. Cardiovasc Eng Technol.

[CR5] Decorato I, Kharboutly Z, Vassallo T, Penrose J, Legallais C, Salsac AV (2014). Numerical simulation of the fluid structure interactions in a compliant patient-specific arteriovenous fistula. Int J Numer Methods Biomed Eng.

[CR6] Delaney EP, Young CN, DiSabatino A, Stillabower ME, Farquhar WB (2008). Limb venous tone and responsiveness in hypertensive humans. J Appl Physiol.

[CR7] Dhar P, Eadon M, Hallak P, Munoz RA, Hammes M (2012). Whole blood viscosity: effect of hemodialysis treatment and implications for access patency and vascular disease. Clin Hemorheol Microcirc.

[CR8] Ferraresso M, Bertoli S, Nobili P, Bortolani EM (2013). Early experience with a newly developed electrospun polycarbonate-urethane vascular graft for hemodialysis access. J Vasc Access.

[CR9] Fry DL (1968). Acute vascular endothelial changes associated with increased blood velocity gradients. Circ Res.

[CR10] Hall RK, Myers ER, Rosas SE, O’Hare AM, Colón-Emeric CS (2017). Choice of hemodialysis access in older adults: a cost-effectiveness analysis. Clin J Am Soc Nephrol.

[CR11] Halliwill JR, Minson CT, Joyner MJ, Monahan KD, Delaney EP, Young CN, Disabatino A, Stillabower ME, William B (1999). Measurement of limb venous compliance in humans: technical considerations and physiological findings. J Appl Physiol.

[CR12] He X, He X, Ku DN, Ku DN (1996). Pulsatile flow in the human left coronary artery bifurcation: average conditions. J Biomech Eng.

[CR13] Hofer M, Rappitsch G, Perktold K, Trubel W, Schima H (1996). Numerical study of wall mechanics and fluid dynamics in end-to-side anastomoses and correlation to intimal hyperplasia. J Biomech.

[CR14] Hoffman J, Jansson J, de Abreu RV, Degirmenci NC, Jansson N, Müller K, Nazarov M, Spühler JH (2013). Unicorn: parallel adaptive finite element simulation of turbulent flow and fluid-structure interaction for deforming domains and complex geometry. Comput Fluids.

[CR15] Hofstra L, Bergmans DC, Hoeks AP, Kitslaar PJ, Leunissen KM, Tordoir JH (1994). Mismatch in elastic properties around anastomoses of interposition grafts for hemodialysis access. J Am Soc Nephrol: JASN.

[CR16] Holzapfel GA, Gasser TC, Stadler M (2002). A structural model for the viscoelastic behavior of arterial walls: continuum formulation and finite element analysis. Eur J Mech—A/Solids.

[CR17] Hu Z, Li Z, Hu L, He W, Liu R, Qin Y, Wang S (2012). The in vivo performance of small-caliber nanofibrous polyurethane vascular grafts. BMC Cardiovasc Disord.

[CR18] Huberts W, Bode AS, Kroon W, Planken RN, Tordoir JHM, van de Vosse FN, Bosboom EMH (2012). A pulse wave propagation model to support decision-making in vascular access planning in the clinic. Med Eng Phys.

[CR19] Karatepe C, Aitinay L, Yetim TD, Dagli C, Dursun S (2013). A novel electrospun nano-fabric graft allows early cannulation access and reduces exposure to central venous catheters. J Vas Access.

[CR20] Lee T, Haq NU (2015). New developments in our understanding of neointimal hyperplasia. Adv Chronic Kidney Dis.

[CR21] Lee T, Qian J, Thamer M, Allon M (2018). Tradeoffs in vascular access selection in elderly patients initiating hemodialysis with a catheter. Am J Kidney Dis.

[CR22] Logg A, Mardal KA, Wells GN (2012). Automated solution of differential equations by the finite element method.

[CR23] Malek AM, Alper SL, Izumo S (1999). Hemodynamic shear stress and its role in atherosclerosis. JAMA.

[CR24] McGah PM, Leotta DF, Beach KW, Aliseda A (2014). Effects of wall distensibility in hemodynamic simulations of an arteriovenous fistula. Biomech Model Mechanobiol.

[CR25] Montini-Ballarin F, Abraham GA, Caracciolo PC (2016) Mechanical behavior of polyurethane-based small-diameter vascular grafts. In: Advances in polyurethane biomaterials. Elsevier, Amsterdam, pp 451–477. 10.1016/B978-0-08-100614-6.00015-910.1016/B978-0-08-100614-6.00015-9

[CR26] Moufarrej A, Tordoir J, Mees B (2016). Graft modification strategies to improve patency of prosthetic arteriovenous grafts for hemodialysis. J Vasc Access.

[CR27] Noordzij M, Jager KJ, Van Der Veer SN, Kramar R, Collart F, Heaf JG, Stojceva-Taneva O, Leivestad T, Buturovic-Ponikvar J, Benítez Sánchez M, Moreso F, Prütz KG, Severn A, Wanner C, Vanholder R, Ravani P (2014). Use of vascular access for haemodialysis in Europe: a report from the ERA-EDTA Registry. Nephrol Dialy Transplant.

[CR28] Predel HG, Yang Z, Segesser LV, Turina M, Buhler FR, Luscher TF (1992). Implications of pulsatile stretch on growth of saphenous vein and mammary artery smooth muscle. The Lancet.

[CR29] Rezakhaniha R, Stergiopulos N (2008). A structural model of the venous wall considering elastin anisotropy. J Biomech Eng.

[CR30] Tordoir J, Canaud B, Haage P, Konner K, Basci A, Fouque D, Kooman J, Martin-Malo A, Pedrini L, Pizzarelli F, Tattersall J, Vennegoor M, Wanner C, Wee PT, Vanholder R (2007). EBPG on vascular access. Nephrol Dial Transplant.

[CR31] USRDS (2017) Chapter 1: Incidence, prevalence, patient characteristics, and treatment modalities, vol 69, no 3. United States Renal Data System, pp S261–S300. 10.1053/j.ajkd.2017.01.020

[CR32] van Haaften EE, Wissing TB, Rutten MC, Bulsink JA, Gashi K, van Kelle MA, Smits AI, Bouten CV, Kurniawan NA (2018) Decoupling the effect of shear stress and stretch on tissue growth and remodeling in a vascular graft. Tissue Eng Part C: Methods 24(7):418–429. 10.1089/ten.tec.2018.0104. arXiv:1011.1669v3 10.1089/ten.TEC.2018.0104 29877143

[CR33] Van Tricht I, De Wachter D, Tordoir J, Verdonck P (2004). Hemodynamics in a compliant hydraulic in vitro model of straight versus tapered PTFE arteriovenous graft. J Surg Res.

[CR34] Wijeyaratne SM, Kannangara L (2011). Safety and efficacy of electrospun polycarbonate-urethane vascular graft for early hemodialysis access: first clinical results in man. J Vasc Access.

[CR35] Yilmaz S (2016). Early experience with a novel self-sealing nanofabric vascular graft for early hemodialysis access. J Vasc Access.

